# Evaluation of Gene Mutations Involved in Drug Resistance in *Mycobacterium Tuberculosis* Strains Derived from Tuberculosis Patients in Mazandaran, Iran, 2013

**Published:** 2014

**Authors:** Farhang Babamahmoodi, Mohammad Reza Mahdavi, Hossein Jalali, Bita Talebi, Payam Roshan, Mehrad Mahdavi

**Affiliations:** 1 Antimicrobial Resistance Research Center, Department of Infectious Diseases, Mazandaran University of Medical Sciences, Sari, Iran.; 2 Thalassemia Research Center, Hemoglobinopathy Institute, Mazandaran University of Medical Sciences, Sari, Iran.; 3 Sina Mehr Research Center, Sari, Iran.

**Keywords:** Tuberculosis, MDR tuberculosis, drug resistance, LPA

## Abstract

Drug resistance (especially multiple drug resistance) in *Mycobacterium tuberculosis* makes global concerns in treatment and control of tuberculosis. Rapid diagnosis of drug resistant strains of the bacteria has vital importance in the prognosis of the disease. The aim of this study was to identify the mutations responsible for drug resistance in *Mycobacterium tuberculosis* strains derived from patients with tuberculosis using line probe assay (LPA) method which rapidly detect drug resistant strains and respective mutations. Sputum samples from tuberculosis patients were collected and cultured on Lowenstein– Jensen medium, and then the colonies of *Mycobacterium tuberculosis* from cultures of 54 bacterial positive cases were randomly chosen for DNA extraction. Bacterial DNA was extracted using standard Cetyl Trimethyl Ammonium Bromide (CTAB) method. In order to identify drug resistant strains and related mutations, LPA method was applied. Three subjects out of 54 investigated cases were resistant to quinolone (5.5%), and resistance to kanamycin/ amikacin, streptomycin, rifampin, and isoniazid were observed in 3 (5.5%), 4 (7.4%), 3 (5.5%), and 2 (3.7%) of the *Mycobacterium tuberculosis* strains, respectively. In the present study, 4 cases (7.4%) were detected to be resistant to more than one drug. Since LPA is a rapid method that simultaneously detects mutations involved in drug resistance, applying this method in the prediction of drug resistance and selecting appropriate treatment in tuberculosis patients is recommended.

Infecting around one-third of the world population, *Mycobacterium tuberculosis* (M-TB) is a serious health threatening pathogen worldwide. 5-10% of the infected patients express the clinical symptoms of tuberculosis (TB) (1). Although TB has been a well known disease since ancient times and despite the advances in medical sciences, large numbers of patients still die due to TB infection (2).For instance, in 2012, 1.1 million people died because of that infection (3). Because of natural selection phenomenon, some strains of these bacteria- resistant to different drugs- have emerged globally. They cause difficulties in the treatment of the disease. 

Strains of M-TB that are simultaneously resistant to at least two different first line drugs- isoniazid, rifampinm pirazinamid and etambotol- used in the treatment of the infection are considered as multi-drug resistant (MDR). The MDR-TB strains are more prevalent in the developing countries (4).

For the first time, in 1990 MDR species of M-TB were reported and since then it has been one of the most important health concerns in many countries. In 2010, 650,000 cases with MDR-TB were identified in the different parts of the world. Afterwards, some species of extensively drug resistant (XDR) and in rare cases, total drug resistant (TDR) TB has been observed (5).

Drug resistant and especially MDR-TB strains raise serious concern in the treatment of TB. Mistreatment of patients with anti-TB drugs is the main cause leading to the incidence of mutations and as a result creation of drug resistance species.

Since M-TB has a low growth rate in culture medium and evaluation of drug sensitivity is expensive and time consuming, after early diagnosis of the disease, the same first line anti-TB drugs is immediately administered for all patients, regardless of their drug sensitivity status (5). Considering this fact, the identification of various drug resistant strains in each region can be helpful in choosing the appropriate strategy for controlling TB and specially MDR strains. This approach was applied for the identification of Pseudomonas aeruginosa drug resistant status in Tehran and Mazandaran province of Iran (6, 7).

LPA (Line Probe Assay) is a DNA test based on DNA amplification by PCR reaction with labeled primers and subsequent hybridization with complementary oligonucleotide probes which are fixed on strip papers. This method has high sensitivity and can evaluate several mutations simultaneously in a single reaction (8).

The identification of various mutations for the prediction of patient’s drug sensitivity status using conventional PCR methods is complicated and time-consuming, while LPA method is a rapid and robust test for achieving that purpose. The aim of this study was to identify the mutations making M-TB resistant to different drugs by applying LPA method among TB patients in Mazandaran province.

## Material and Methods

In the present study, the colonies of M-TB from 54 samples were randomly selected. Before culturing sputum on Lowenstein–Jensen medium, all samples were treated with 4% NaOH.

Bacterial genomic DNA was extracted from two colonies using standard cetyl trimethyl ammonium bromide (CTAB) method.

In order to detect 11 different mutations involved in resistance to 5 different drugs, line probe assay (LPA) method was applied. The test was done using a commercial kit (GenID, Germany) which employed PCR/ hybridization technique. In this method, at first, fragments of the bacterial genes were amplified by two separate multiplex PCR reactions. Then, the PCR products were denatured and hybridized with their complementary probes coated on specific locations on strips. After staining procedure, the presence of mutations was evaluated by analyzing the pattern of bands created on the strips.

## Results

In the present study, 54 TB patients of Mazandaran province (including 33 males and 21 females) were selected. Eleven subjects (9 females and 2 males) were resistant to at least one drug. Streptomycin resistant strains of M-TB had the highest incidence rate in the region (7.4% of all cases). Resistance to quinolone, kanamycin/ amikacin and rifampin drugs had the same frequencies (5.5%), and isoniazid resistant strains were less common (1.4%) (Table 1).

20.4% of the cases were resistant to at least one of the investigated drugs and in 7.4% of subjects, more than one mutation (each responsible for resistance to different drugs) was observed. All isoniazid resistant cases carried the same mutation in *kat G* gene and no subject was identified with mutation in *inhA* gene. In cases detected as resistant to rifampin, the related mutation was observed at codon 516 of *rpoB* gene. Mutations at positions 1401 and 1483 of *rrs *gene that are responsible for kanamycin/ amikacin resistance had the same frequencies. Among the strains of M-TB considered as streptomycin resistant, only mutation in *rpsL* gene was detected and no mutation in *rrs *gene was identified. One of the studied patients was co- infected with TB and HIV (Table 2).

## Discussion

Inappropriate treatment of infected patients is the main cause of spreading the disease, in particular in the developing countries. Prior to treatment of the disease, it is necessary to diagnose the disease and identify drug resistant strains as fast as possible. The current study evaluated the presence of 11 different mutations involved in the emergence of resistant strains of M-TB in TB patients using LPA method.

**Table 1:** Frequency of gene mutations causing drug resistant TB, Mazandaran, 2012

Drugs	Isoniazid		Rifampin			Streptomycin			Kanamycin/ Amikacin		Quinolone
Mutation	inhA -16-8-15	katG 315	rpoB 516	rpoB 531	rpoB 526	Rrs 522-526	rpsL 43	rpsL 88	Rrs 1483	rrs 1400-1401	gyrA 90-94
Mutations in male subjects	2	3	3	1	2	0	0	0	3	2	0
Mutations in female subjects	1	0	0	1	2	0	0	0	0	0	0
Total Observed mutations	3	3	3	2	4	0	0	0	3	2	0
Number of drug resistant patients	3		3			4			3		2

**Table 2:** frequency of patients with resistance to one or more drugs

Type of drug resistance	Drug names	Number of cases with mutations	Total
Single drug resistance	rifampin(*rpoB *516 mutation)	2	7(12.9%)
	kanamycin	2	
	streptomycin (*rpsL*)	1	
	isoniazid (*katG*)	2	
Double drug resistance	stereptomycin + qinolon	3	4 (7.4%)
	rifampin + kanamycin	1	
Multiple drug resistance	At least to isoniazid and rifampin	0	0

Various mutations in different genes have been introduced responsible for resistance to a variety of medications in M-TB. In this study, the most frequent mutations involved in drug resistance were examined. It was observed that mutations in* inh A and kat G *genes are related to resistance to isoniazid and resistance to rifampin is caused by mutations in *rpo B* gene. *Rrs*, *rpsL *and *gydB* gene mutations make the bacillus resistant to streptomycin and resistance to kanamycin/ amikacin is induced by mutations in* rrs* and *eis* genes. Moreover, it was reported that *rrs* and *tylA *gene mutations lead to resistance to kapromycin. *PncA* and *gyrA* gene mutations are responsible for resistance to pirazinamid and quinolone, respectively (9).

Sensitivity and specificity of the mutations on the abovementioned genes were studied in different studies and these positions are well known as common mutations responsible for M-TB drug resistance. In two separate studies in South Korea and Brazil *katG*-315*, inhA*-15*, rps*-l43*, rrs*-1401 and* rpoB*-531were introduced as common mutations (10, 11). Isakova et al. in 2005 reported that positions 531, 516 and 536 of *rpoB* genes are the common sites of mutation and mutation at position 531 is more frequent than mutations in other positions of this gene (12). Another study in South Korea in 2013 showed that *gyrA*-94, *embB*-306 and *pncA*-159 positions are hot spots for mutations (10).

In Venezuela, mutations in *katG* had 88.2% sensitivity and 100% specificity (13). In Iran, and in Ahvaz, different mutations in *inhA*, *rpoB* and *katG* genes were detected in M-TB strains from the patients. In Zanjan (a central province of Iran) mutations in *katG*-315, *rpoB*-531 and *inhA*-15 positions were shown to have high incidence rate in isoniazid resistant strains of bacteria derived from TB patients (14). Mutations on *gyrA* and *rrs* genes in Tehran were detected in another study (15). Doustdar et al. in 2007 investigated the presence of mutations at positions 531, 516 and 526 of *rpoB *gene and indicated that the identification of these mutations could help physicians to diagnose resistance to rifampicin (16). These mutations are almost common in Iran while other mutations in this gene are rarely reported. They stated that comparing to the other parts of the world, mutations at positions *rpoB*-516 and *rpoB*-526 have different frequencies.

In Golestan province (located at North of Iran), Javid et al. reported that in 87 studied cases 5.7% and 3.4% had* katG* and *inhA* gene mutations respectively and 4.6% of subjects had a mutation in *rpoB* gene (17). In that study, two cases had mutations in *katG* and* inhA* genes, simultaneously. In 2001, Poorhaji et al. in Mazandaran reported that among the patients with isoniazid resistant TB, mutation in *katG* gene has the highest frequency (18). As some of the evaluated mutations in our study, were not previously investigated in the North of Iran, the present study gives a genuine and more comprehensive knowledge about the frequency of these DNA changes in Mazandaran province.

Similar to the reports in Golestan and Azerbaijan provinces, in our study, no subject with MDR-TB was detected. Making these reports and our study into consideration, MDR-TB is not a serious problem in Iran (17). The present study is quite comparable with other studies in which mutation in *katG* gene is responsible for resistance to isoniazid in most of the TB patients in Iran and similar to the Khalilzadeh et al.’s report, in the present investigation rifampicin resistant strains were more frequent than isoniazid resistant strains (22). Zaker Bostanabadi et al. reported that mutation at position 526 of *rpoB* gene is related to rifampin resistance in Iran while in our study only mutation at position 516 of *rpoB* gene was detected (19). It is important to mention that mutation at position *rpoB*-526 was rarely reported (20, 21).

We observed resistance to second line drugs used in the TB treatment in some cases. This issue is a risk factor for the treatment of MDR-TB patients and this will cause difficulties if it becomes widespread. Second line drugs are used for confronting tuberculosis when a patient is resistant to first line drugs thus, resistance to second line drugs lead to no responsiveness to treatment in MDR-TB patients and the spread of the MDR-TB.

32.7% of evaluated patients were sputum smear-negative for TB while the M-TB was grown on Lowenstein–Jensen medium. Since LPA can quickly and easily predict the presence of M-TB and its drug resistance status and because hand getting results through sputum culture is time consuming, we recommend LPA method for the early diagnosis of TB and the identification of drug resistant strains.

## Conflict of interest

The authors declared no conflict of interests.
